# A Preliminary Analysis of Hearing Loss and Its Association With Mild Cognitive Impairment

**DOI:** 10.1093/geroni/igaa057.1461

**Published:** 2020-12-16

**Authors:** Mary Caroline Yuk, Rebecca Allen, Marcia Hay-McCutcheon, Dana Carroll, Anne Halli-Tierney

**Affiliations:** 1 The University of Alabama, Tuscaloosa, Alabama, United States; 2 Alabama Research Institute on Aging, Tuscaloosa, Alabama, United States; 3 Audiology, Tuscaloosa, Alabama, United States; 4 Auburn University Harrison School of Pharmacy, Tuscaloosa, Alabama, United States

## Abstract

Age related hearing loss, or presbycusis, is a global condition that is increasing in its prevalence. Despite being one of the most common chronic conditions among the older population, there is much more to understand about its association with other aspects of physical and emotional health and well-being. Current research is suggesting that hearing loss is more prevalent in those with cognitive impairment compared to those without cognitive impairment. This study analyzed the incidence of hearing loss and its linkage to mild cognitive impairment in a community-dwelling geriatric population. With the increasing prevalence of this condition in both rural and urban communities of Alabama, it becomes a more pressing matter to understand comorbidities and risk factors for future decline in functioning. This study was conducted in an interdisciplinary geriatrics primary care outpatient clinic in a Family, Internal, and Rural Medicine department affiliated with a university medical center in the Deep South. Ninety-one participants completed the Montreal Cognitive Assessment (MoCA) and a hearing screening. Hearing screenings were conducted in quiet rooms in the medical center using Phonak hearing screening cards. Detection of 500, 1000, 2000, and 4000 Hz tones was assessed. Pearson correlation analyses demonstrated an association between hearing loss mild cognitive impairment. Poorer hearing was significantly associated with lower scores on the MoCA. Conducting behavioral health screenings like this in other primary geriatrics clinics and community settings could improve care and identification of patient needs by integrating important data regarding comorbidities and independent living.

